# Microstomia Release in Post-burn Contractures: A Case Report

**DOI:** 10.7759/cureus.79184

**Published:** 2025-02-17

**Authors:** Mohd Altaf Mir, Sakshita Pal, Juhi Sharma, Divakar Goyal

**Affiliations:** 1 Burns and Plastic Surgery, All India Institute of Medical Sciences, Bathinda, Bathinda, IND; 2 Anesthesiology, Pain Medicine, and Critical Care, All India Institute of Medical Sciences, New Delhi, New Delhi, IND; 3 Anesthesiology, All India Institute of Medical Sciences, Bathinda, Bathinda, IND; 4 Trauma and Emergency, All India Institute of Medical Sciences, Bathinda, Bathinda, IND

**Keywords:** commissuroplasty, decreased mouth opening, facial burns, microstomia, post-burn microstomia

## Abstract

Microstomia following orofacial and neck burns with subsequent contractures poses a significant challenge to the anesthesiologist as well as the plastic surgeon. Fibrosed external nares, microstomia, and contracture bands make a difficult airway. Deformed facial structure and raw area of burns make ventilation difficult. One of the significant causes of morbidity and mortality under anesthesia is the “cannot intubate, cannot ventilate” scenario. Further reconstruction of oral commissures in such cases is always challenging because it provides good functional and acceptable aesthetic results. Here, we present a case of microstomia having limited mouth opening in which fiberoptic nasal intubation and bilateral oral commissuroplasty were performed for microstomia release. In our case, we achieved mouth opening, which was preoperatively 15 mm to 55 mm postoperatively using Dieffenbach commissuroplasty.

## Introduction

Facial deformities like microstomia are often viewed as one of the most distressing types of disfigurement due to the importance of the mouth in both appearance and function. Microstomia is defined as the narrowing of the oral aperture due to contraction of the lateral commissures following burns or trauma of the perioral region. Tissue heals with secondary intention with scar formation and contracture formation, leading to a decreased ability to open the mouth. Scar and contracture formation decreases the normal functioning of the orbicular muscle [1].

The mouth, lips, and perioral area have significant roles in facial expression, speech, eating, whistling, sucking, and providing oral competence. Thus, the sequelae of microstomia include malnutrition, respiratory and speech difficulties, risk of aspiration, poor oral hygiene, difficulty with future endotracheal intubation, and cosmetic concerns. It also causes significant emotional distress. This is because any deformities of the face have always been considered one of the least desirable handicaps [2].

The anesthetists often face challenges in intubating these cases; however, fiberoptic intubation has made it easy for anesthetists to manage these complex cases [3,4].

Several methods of microstomia reconstruction have been mentioned in the literature. In this article, we are describing a case of severe post-burn microstomia managed by bilateral commissuroplasty. We used the Dieffenbach bilateral commissuroplasty because it is distinctive for its straightforward approach to commissure reconstruction, effectively addressing the lateral edges of both the upper and lower lips. It stands out as superior to other commissuroplasties due to its simplicity and effectiveness in reconstructing the commissure.

Reconstructive techniques for microstomia vary, depending on the severity of the condition. The article mentions bilateral commissuroplasty, a surgical procedure often used to treat severe microstomia, particularly post-burn cases. The Dieffenbach bilateral commissuroplasty is highlighted as a practical approach for addressing the lateral edges of both the upper and lower lips, making it especially suitable for commissure reconstruction. This method is considered superior to other types of commissuroplasty due to its simplicity and efficiency in restoring the mouth's ability to open and function properly. The goal of this procedure is not just aesthetic but also functional, improving the patient's ability to speak, eat, and maintain oral hygiene after suffering from severe tissue contracture [5-9].

## Case presentation

**Figure 1:**
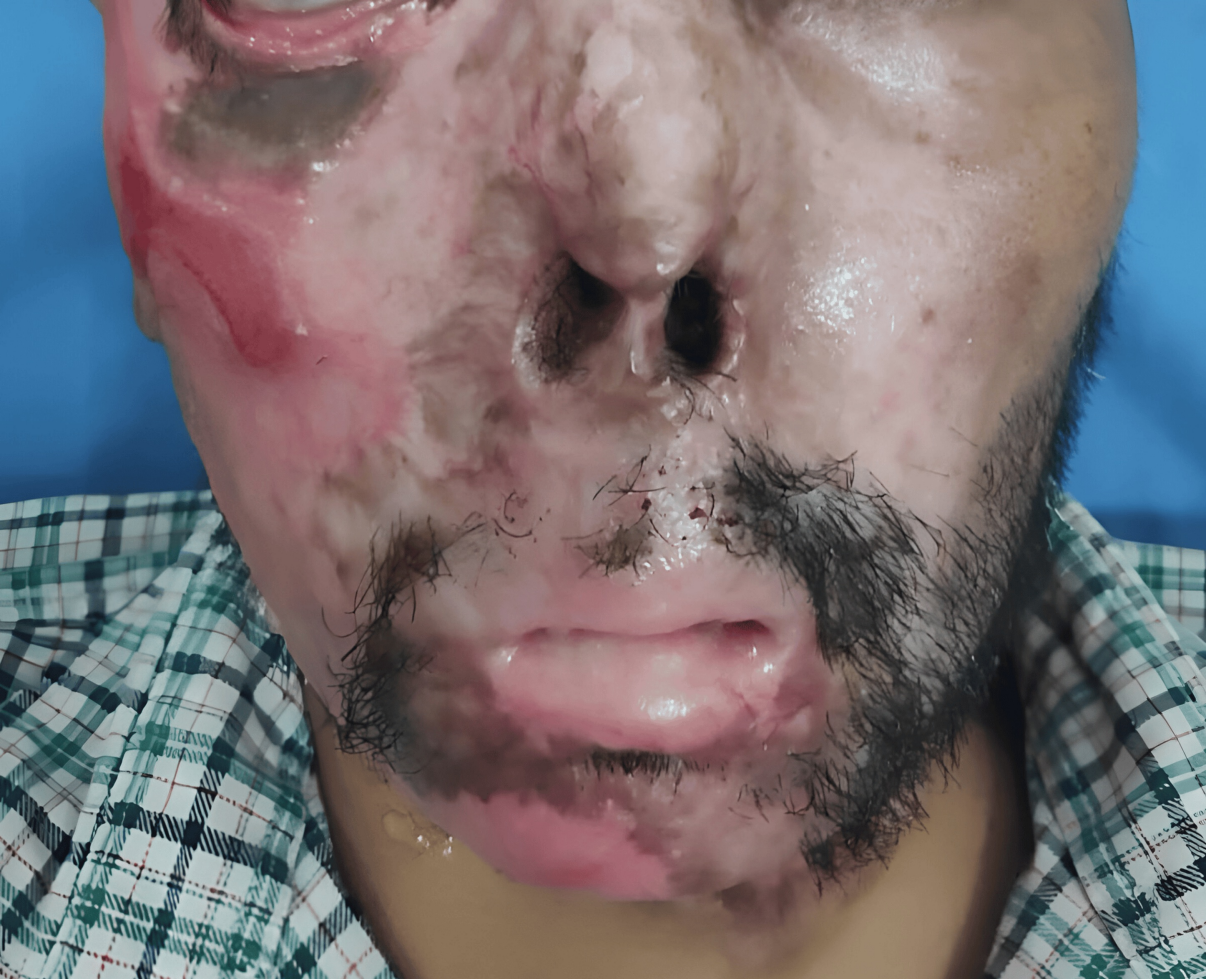
Preoperative view Preoperative view at three months post-burn injuries showing microstomia measuring 25 mm with right eye ectropion and nasal deformity. Written consent was obtained from the patient for the images and their publication.

A male patient in his late 30s was admitted to the hospital with complaints of extensive facial and neck burns from hot water following a seizure episode two months back. He sustained extensive facial burns involving the right eye, external nares, mouth, and anterior neck. He developed right lower eyelid ectropion and post-burn microstomia with deformed external nares. The microstomia interfered with enteral intake and communication. The patient had been drinking liquids with difficulty with feeding jejunostomy in situ for the past four months. The patient had a history of seizures for the past two and a half years, for which he was on regular medication. He was not able to open his mouth completely. Oral hygiene was deplorable. The clinical examination was done, and the distance between the two oral commissures was around 2.5 cm (Figure 1).

The philtrum column was lost and replaced by early scarring in two months. This also made the surgery easy to raise the flap.

There was a scar on the right side of the face post-burn injury with a small raw area at the zygomatic region. In the anesthetic check-up clinic, a preoperative airway assessment was done, which revealed a mouth opening of less than 15 mm due to burns. The temporomandibular joints were not subluxated. Preoperative temporomandibular joint movements were clinically checked, and intraoperatively after surgery, temporomandibular movements in the full range were achieved using a heister device. The thyromental distance was less than two fingers in breadth. Neck extension was restricted due to a contracture band on the anterior part of the neck. The patient was counseled for awake fiberoptic nasal intubation and explained the need for a tracheostomy if awake fiberoptic nasal intubation is unsuccessful.

Management

On the day of surgery, the patient was adequately fasted. Airway preparation was done with nebulization, trans-tracheal block using 4 ml of 2% lignocaine, and superior laryngeal nerve block with 4 ml of 2% lignocaine bilaterally. A 7 mm nasal airway coated with lignocaine jelly was inserted to lubricate and check the patency of the nasal airway. Also, 0.2 mg of intravenous glycopyrrolate was administered. A 6.5 mm internal diameter endotracheal tube (ETT) was railroaded over the fiberoptic laryngoscope.

After proper positioning, a well-lubricated fiberoptic scope was inserted through the left nostril. The trachea was visualized, however, with some difficulty. This was probably due to some scarring and traction of tissue leading to internal anatomical changes. Once the carina was visualized, the ETT was inserted into the trachea and connected to the ventilator. Bilateral air entry and capnograph were confirmed, and the patient was anesthetized.

Procedure

**Figure 2:**
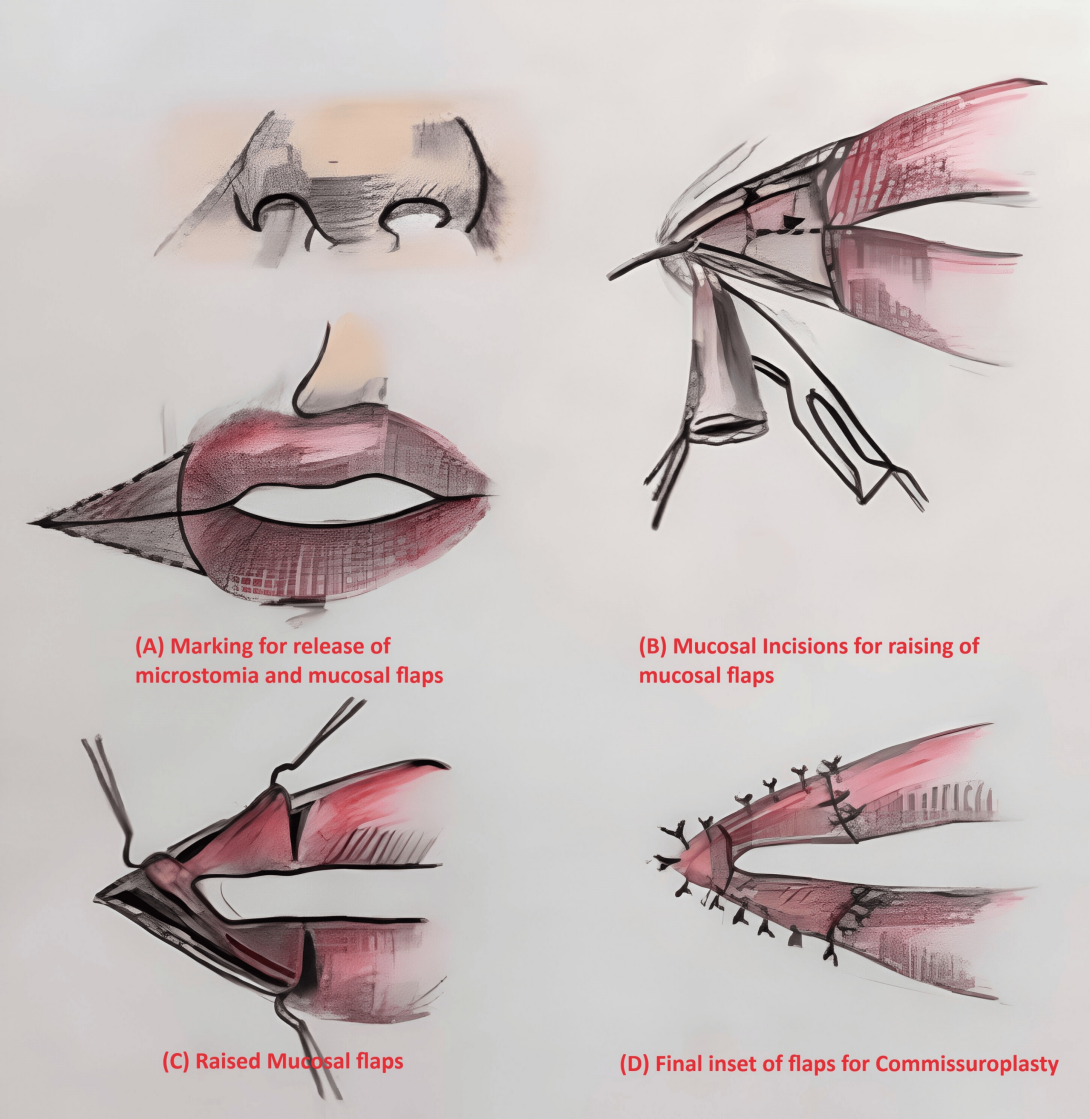
Artistic diagram This figure describes Y-V mucosal advancement flaps originally described by Dieffenbach. Marking for new oral commissure reconstruction as depicted in (A). A fibrous triangle of tissue with a post-burn scar was excised, making a straight Y limb incision and leaving the oral mucosa and orbicularis oris as depicted in (B). A V incision was given over the mucosal side to advance as the Y-V advancement flap led to the formation of three flaps - superior, middle, and inferior - as depicted in (C). Superior and inferior flaps were used to reconstruct the vermillion, and the middle flap, which was the lateral flap, was used to reconstruct the angle of the mouth. The middle flap was then advanced and sutured to the newly planned commissure site on both sides as depicted in (D). Credit: Illustration by author SP.

We have chosen the Y-V mucosal advancement flap method originally described by Dieffenbach (Figure 2).

**Figure 3:**
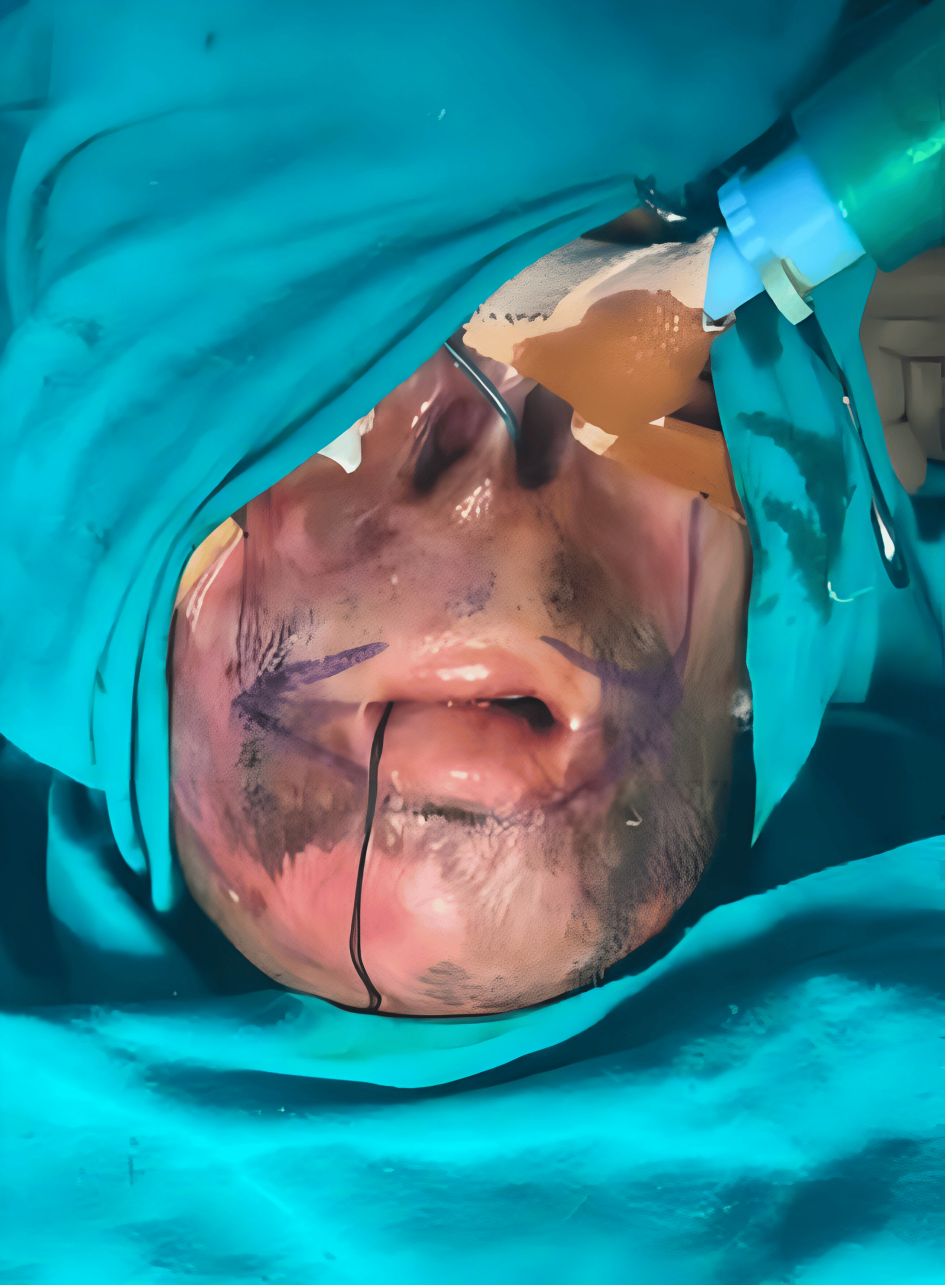
Intraoperative view The mid-pupillary axis and horizontal line from both the oral commissures were used to determine the new lateral oral commissure location.

Marking was done (Figure 3) to determine the new oral commissure. A horizontal line was marked at the level of forming of both the oral commissures and was increased to both sides. A vertical line was dropped from mid-pupil up to the level of the horizontal line. Crossing points of both lines were marked as new places for commissures on both sides.

Dieffenbach (Y-V advancement flap) was marked on both sides of the commissure. A fibrous triangle of tissue with a post-burn scar was excised, and a straight Y limb incision was made, leaving the oral mucosa and orbicularis oris (Figure 2).

**Figure 4:**
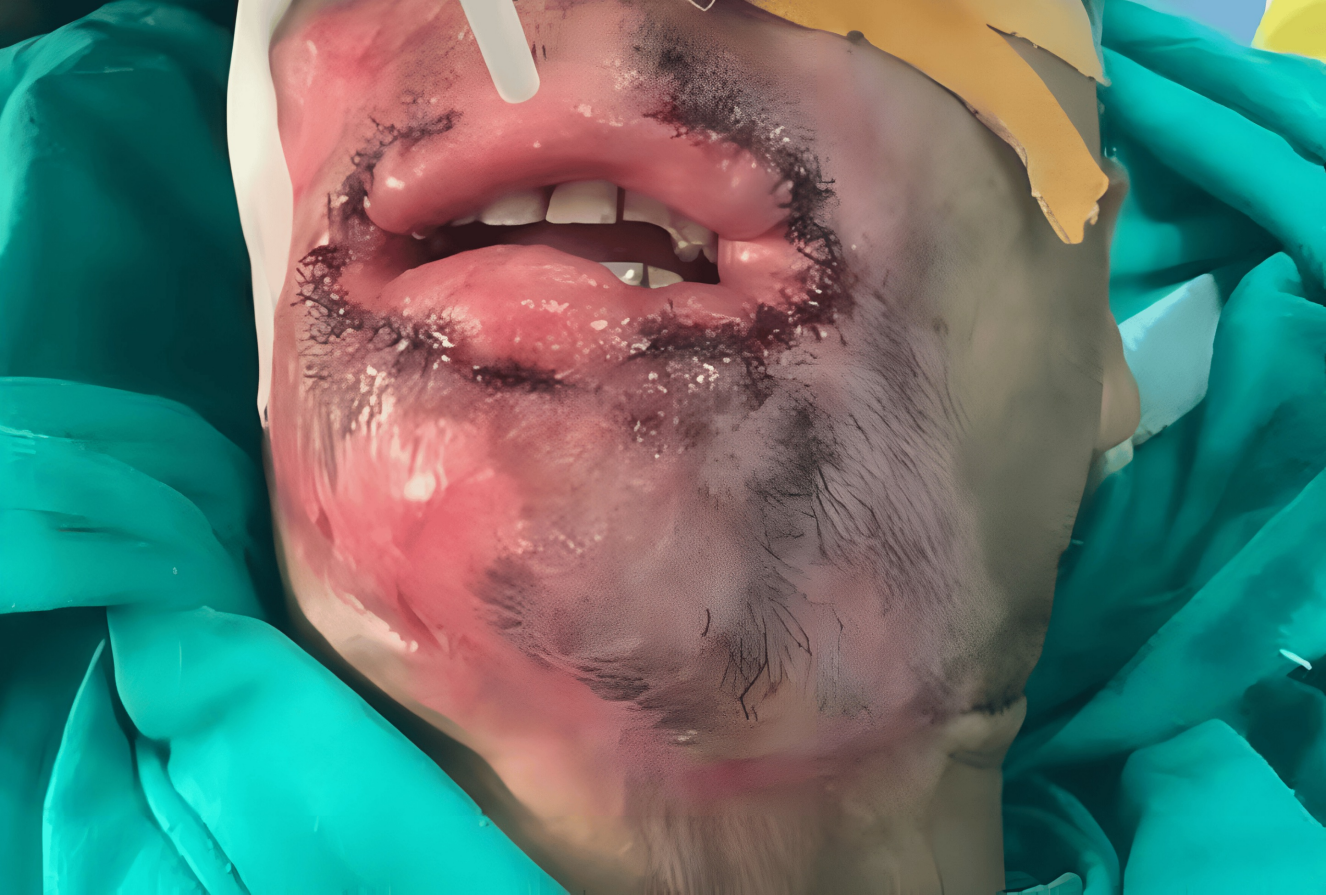
Intraoperative view Intraoperative view showing suturing of the flaps and formation of new oral commissure.

Orbicularis oris is responsible for the competence of the mouth, so it should be preserved during dissection. A V incision was given over the mucosal side to advance as the Y-V advancement flap (Figure 2), forming three flaps - superior, middle, and inferior. Superior and inferior flaps were used to reconstruct the vermillion, and the middle flap, which was the lateral flap, was used to reconstruct the angle of the mouth. The middle flap was advanced and sutured to the new planned commissure site on both sides (Figures 2, 4).

5-0 Vicryl suture was used for suturing.

The patient was kept postoperatively under observation for 10 days. The patient was fed with Ryle’s tube for three days. After 10 days of healing, the patient could take liquids and soft food orally, and the jejunostomy tube was removed. At three weeks, the knots of Vicryl sutures were removed.

Outcome and follow-up

**Figure 5:**
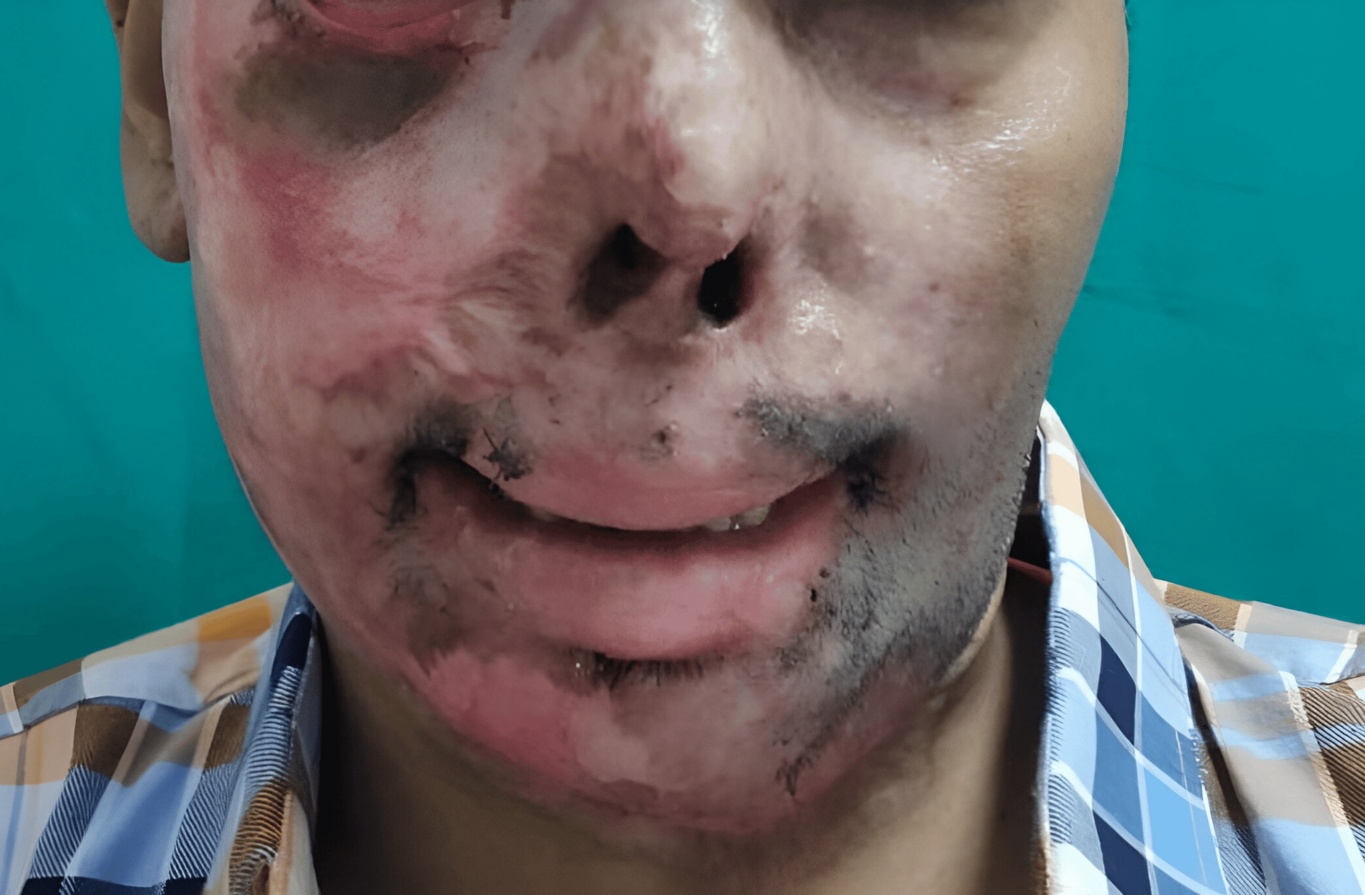
Postoperative view At three weeks postoperatively, with an inter-commissural distance of 64 mm. Written consent was obtained from the patient for the images and their publication.

The distance between two oral commissures was around 25 mm in the preoperative clinical examination. In the postoperative period, the distance between oral commissures was 64 mm (Figure 5).

There was a significant improvement in mouth opening after microstomia surgery. The patient could take semisolid and solid food after three weeks of surgery and speak. He had partial competence in oral aperture, and pursing exercises were recommended. Ectropion release and nasal reconstruction will be done after months.

## Discussion

Anesthetic management

Anesthetic management in patients with burns presents multiple challenges. Anesthesiologists need in-depth knowledge of the implications of acute and chronic burns and airway management in both scenarios. A timely and planned intervention and teamwork are the keys to safe and successful management.

Anesthetic management for potentially difficult airways includes airway assessment, resource mobilization, airway control, monitoring, and aftercare [3]. Our patient had microstomia along with a slight fixed flexion deformity of the neck due to the small contracture band in the anterior neck. This made the conventional direct laryngoscopy almost impossible. Since our patient required nasal intubation because of commissurotomy, we planned awake nasal fiberoptic intubation. Our next plan, had intubation failed, was tracheostomy by the surgeons since the contracture band was small and tracheal landmarks were palpable.

Fixed flexion deformity, common in patients with burns, poses excellent difficulty securing the airway due to non-alignment of the oro-naso-laryngopharynx. An awake fiberoptic bronchoscope (FOB) intubation has been the gold standard practice for securing the airway. However, many studies have documented the failure to guide the ETT despite clear visualization of the carina. This can be attributed to the acute angulation due to flexion deformity. Hence, various techniques, including laryngeal mask airway (LMA)/intubating laryngeal mask airway (ILMA), aiding successful intubation, have been attempted and achieved ventilation [4].

Another key tool in patients with limited neck extension and adequate mouth opening is video laryngoscopes. However, it should be noted that although laryngeal visualization is easy, differences between the visual axis of the camera and tube insertion may not be aligned in a flexed neck. Other well-known and documented techniques are tumescent anesthesia or contracture band release under local anesthesia using ketamine followed by intubation.

In our case scenario, neck extension was restricted due to the contracture band. However, it was not severe. The patient was planned for commissurotomy due to microstomia, hence mandating nasal intubation. We, therefore, went ahead with awake nasal fiberoptic intubation. Had our plan failed, we had surgeons on standby for tracheostomy under local anesthesia.

Another point we would want to emphasize is the possibility of difficulty recognizing internal landmarks while performing nasal intubation. This occurs due to internal tissue scarring and external deformity, which makes the procedure difficult, as in our case. Edematous tissues and the increased possibility of bleeding further complicate this. A well-lubricated fiberoptic scope and small-sized ETT are recommended.

Surgical management 

Various surgical treatments are described in the literature. Microstomia cases are not so common. It is a rare complication following burn injuries and is seen in around 3.7% of cases of thermal injuries [5]. This makes the management of microstomia challenging. Oral commissure and modiolus have complex anatomy, so management of microstomia needs proper planning and execution. Microstomia may be managed initially by conservative, non-operative methods like prosthetics and physiotherapy [6]. Orthotic appliances are essential in the non-operative management of microstomia, providing adequate support during the healing process. Available in both removable and fixed forms, these devices help establish a comfortable resting bite for the patient, ensuring symmetry of the oral commissures and stabilizing the orbicularis oris muscle through two-point fixation. By counteracting wound contraction, orthotic appliances play a pivotal role in preventing further narrowing of the oral aperture, making early implementation crucial for optimal treatment outcomes [7]. Examples of orthotic appliances used to manage microstomia include the Modified Dynamic Mouth Splint, Molt Mouth Prop, Tooth-Borne Removable Splint, Oral Commissure Expansion Prosthesis, and Dynamic Intraoral Devices, among others [8]. Among these, the Modified Dynamic Mouth Splint is the most effective option, as it provides multidirectional stretch - horizontally, vertically, and circumferentially - while also being user-friendly. Its comprehensive stretch and ease of use make it a preferred choice in clinical settings for managing microstomia effectively [9].

Non-operative management may be considered as initial therapy in facial burns, given the ability of the face to heal secondary to its robust blood supply [10]. Splintage with regular oral exercises and massages is the most common therapy [11]. However, such therapies have a high chance of recurrences. Moreover, in non-compliant patients, we cannot produce good results with such therapies, which is the most common cause of failure of such therapies [10,11]. Thus, surgical management is the mainstay treatment. Operative management aims to provide mucosal lining and to recreate the functional oral sphincter of the orbicular muscle [10,11].

In 1831, Dieffenbach described his technique for microstomia. In his technique, he first excised the triangular scar area near the oral commissure, then reconstructed the oral commissure with the help of the advancement of superior, inferior, and lateral mucosal flaps [12]. This procedure offers easy commissure reconstruction where the lateral edges of the upper and lower lips get covered, which is why we preferred it over others and used it in our case to correct microstomia. Converse modified this technique and used a trilobed advancement flap to reconstruct the oral commissure in severe cases [13]. This technique effectively eliminates the appearance of a narrow mouth and, to a limited extent, allows a greater degree of mouth opening. Further modified by Gillies and Millard, and later by Johns et al., a method was developed using a vermilion flap from the corner of the mouth to reconstruct the upper lip. This method involves advancing the oral mucosa from the inner aspect of the lower lip to create a new vermilion border, effectively covering the deficient area in the lower lip [14,15]. Many surgical techniques are available for correcting microstomia, including commissurotomy, Z-plasty, W-plasty, and various mucosal advancement flaps. Skin grafting methods, such as split-thickness, full-thickness skin grafts, and mucosal grafts, are also commonly employed. For more extensive cases, skin-muscle grafts and vestibuloplasty may be utilized. Lip or commissure augmentation can also be considered. Still, this technique is mainly for individuals with congenital microstomia or those with a reduced oral aperture due to previous surgical resections and reconstructions. Further, it is also seen that the dental material used for taking dental impressions is usually moldable in any shape. It can be used in any shape in the early postoperative period after healing is complete to maintain the inter-labial distance of the commissure [16].

Patient’s perspective

I had burn injuries a few months ago. The incident was harrowing and unpleasant. To add to my miseries, I had a problem in the right eye; the everted lower eyelid irritated me very much. I also had injuries on my nose and mouth, leading to reduced mouth opening, which made it difficult for me to speak and eat; because of this, a tube was inserted in my stomach, through which fluids were given to me. I went to the eye doctor first because I had vision problems that were troubling me more. But from there, I was referred to a plastic surgeon to treat reduced mouth opening. Here, the doctors explained the whole procedure, and though I was scared about it, I gave my consent, and I don’t regret that decision. After the operation, the initial few days were difficult, but later, with continuous exercises as recommended by the physiotherapist and the removal of sutures, I regained my oral function. I was able to eat soft foods and talk a bit. This operation was a life changer for me.

Learning points

This case highlights the anesthetic management of a burn patient with submental and perioral contracture, requiring a careful approach to managing a difficult airway under anesthesia. The use of an awake nasal fiberoptic bronchoscope allows for secure airway management in such challenging situations. Additionally, the case demonstrates the correction of microstomia using the Dieffenbach method, a rare but significant complication of burn injuries. One of the key advantages of this technique is its minimal dissection, which helps improve oral intake and alleviates difficulties with speech, breathing, and cosmetic appearance. With the successful correction of microstomia, the patient can now undergo oral intubation for further surgical interventions, including correction of right eye ectropion and nasal deformity.

## Conclusions

Microstomia is a challenging condition characterized by the narrowing of the mouth opening, often caused by burns or trauma. This limitation impairs essential functions like eating, speaking, and breathing, leading to difficulties with oral hygiene, malnutrition, and speech problems. Additionally, the emotional impact of facial disfigurement can be profound, affecting self-esteem and social interactions. Surgical reconstruction, such as Dieffenbach commissuroplasty, is a key treatment that addresses the deformity by widening the mouth opening. This procedure improves the aesthetic appearance and restores essential functions, offering significant relief and improving the quality of life for affected individuals.
